# Mucosal Regulatory T Cells and T Helper 17 Cells in HIV-Associated Immune Activation

**DOI:** 10.3389/fimmu.2016.00228

**Published:** 2016-06-20

**Authors:** Pushpa Pandiyan, Souheil-Antoine Younes, Susan Pereira Ribeiro, Aarthi Talla, David McDonald, Natarajan Bhaskaran, Alan D. Levine, Aaron Weinberg, Rafick P. Sekaly

**Affiliations:** ^1^Department of Biological Sciences, School of Dental Medicine, Case Western Reserve University, Cleveland, OH, USA; ^2^Department of Medicine, Division of Infectious Diseases, University Hospitals, Case Western Reserve University, Cleveland, OH, USA; ^3^Department of Pathology, Case Western Reserve University, Cleveland, OH, USA; ^4^Department of Microbiology and Molecular Biology, School of Medicine, Case Western Reserve University, Cleveland, OH, USA; ^5^Department of Pharmacology, School of Medicine, Case Western Reserve University, Cleveland, OH, USA

**Keywords:** T_regs_, Th17, HIV, mucosal immunity, Foxp3

## Abstract

Residual mucosal inflammation along with chronic systemic immune activation is an important feature in individuals infected with human immunodeficiency virus (HIV), and has been linked to a wide range of co-morbidities, including malignancy, opportunistic infections, immunopathology, and cardiovascular complications. Although combined antiretroviral therapy (cART) can reduce plasma viral loads to undetectable levels, reservoirs of virus persist, and increased mortality is associated with immune dysbiosis in mucosal lymphoid tissues. Immune-based therapies are pursued with the goal of improving CD4^+^ T-cell restoration, as well as reducing chronic immune activation in cART-treated patients. However, the majority of research on immune activation has been derived from analysis of circulating T cells. How immune cell alterations in mucosal tissues contribute to HIV immune dysregulation and the associated risk of non-infectious chronic complications is less studied. Given the significant differences between mucosal T cells and circulating T cells, and the immediate interactions of mucosal T cells with the microbiome, more attention should be devoted to mucosal immune cells and their contribution to systemic immune activation in HIV-infected individuals. Here, we will focus on mucosal immune cells with a specific emphasis on CD4^+^ T lymphocytes, such as T helper 17 cells and CD4^+^Foxp3^+^ regulatory T cells (T_regs_), which play crucial roles in maintaining mucosal barrier integrity and preventing inflammation, respectively. We hypothesize that pro-inflammatory milieu in cART-treated patients with immune activation significantly contributes to enhanced loss of Th17 cells and increased frequency of dysregulated T_regs_ in the mucosa, which in turn may exacerbate immune dysfunction in HIV-infected patients. We also present initial evidence to support this hypothesis. A better comprehension of how pro-inflammatory milieu impacts these two types of cells in the mucosa will shed light on mucosal immune dysfunction and HIV reservoirs, and lead to novel ways to restore immune functions in HIV^+^ patients.

## Systemic Activation and Gut Mucosal Dysbiosis in HIV Disease

Human immunodeficiency virus (HIV) associated systemic immune activation constitutes persistent immune dysfunction associated with chronic non-infectious events that include cardiovascular, hepatic, and renal disease, as well as non-AIDS malignancies in cART-treated patients (1–3). Immune reconstitution inflammatory syndrome (IRIS) also comprises of immune abnormalities, although in patients with advanced immunodeficiency and underlying opportunistic infections (4, 5). While cART has dramatically changed the fatal course of the epidemic in HIV-infected patients, continuous treatment poses significant challenges in terms of costs and clinical safety. Moreover, despite profound reductions in acute opportunistic infections, persons with treated HIV infection are surviving to experience residual inflammation and HIV-associated chronic end-organ diseases. Antiretroviral therapy also has variable effects in terms of completely reconstituting immune functions. Patients who do not respond to cART completely, i.e., the immunologic non-responders (INR), can maintain much lower peripheral CD4^+^ T-cell counts (e.g., <350 cells/μl), despite durable suppression of plasma viral loads to undetectable levels for many years. In patients successfully responding to cART (immune responders; IR; defined variably as CD4 >500 cells/μl), residual disease manifests as milder inflammation and immune senescence. In both settings, the persistence of viral reservoirs in latently infected cells in adipose and lymphoid tissues are evident (2, 3, 6–12). These reservoirs contribute to rapid rebound of virus replication upon cART termination. Thus, even in the cART era, persistent immune dysregulation predisposes patients to AIDS and non-AIDS clinical events, and also may be linked to persistence of HIV.

Ongoing HIV replication, microbial translocation products, and co-infections have been shown to stimulate the expression of type-1 interferons (IFNs) as well as other pro-inflammatory cytokines in immune cells in blood and in lymphoid tissues (6, 9, 10, 13–15). These cytokines accelerate residual disease progression by promoting effector CD4^+^ cell activation and increasing the pool of cells permissive to HIV-1 infection in lymph nodes and mucosal lymphoid tissues (16). Furthermore, loss of Th17 cells and breaches in gut epithelial barriers facilitate increase in systemic levels of bacterial products, prompting persistent immune activation and HIV reactivation (6, 17), and independently predicting mortality in HIV patients and simian immune-deficiency (SIV)-infected rhesus macaques (9, 18–20). While early cART treatment can reverse to a significant extent the HIV-inflicted gut mucosal injury (20, 21), restoration of CD4^+^ T cells in gut lamina propria is minimal to incomplete, when compared to findings in uninfected controls (15, 22–28). While these studies begin to further our understanding of gut mucosal dysbiosis (6, 9, 17, 29–35), to date, most of the research on HIV-dependent immune activation has been derived from analysis of circulating T cells (21, 36–40). Further studies are required to examine regional or local effects of inflammation and inflammatory products, as well their site-specific effects on mucosal CD4^+^ T cells and HIV reservoirs. Because mucosal Th17 cells and regulatory T cells (T_regs_) are critical determinants of microbial translocation and inflammation, it is important to study the precise interactions between these cells and various parameters of systemic immune activation.

## Immune Activation Correlates to Increased HIV Reservoir Size

**Figure 1:**
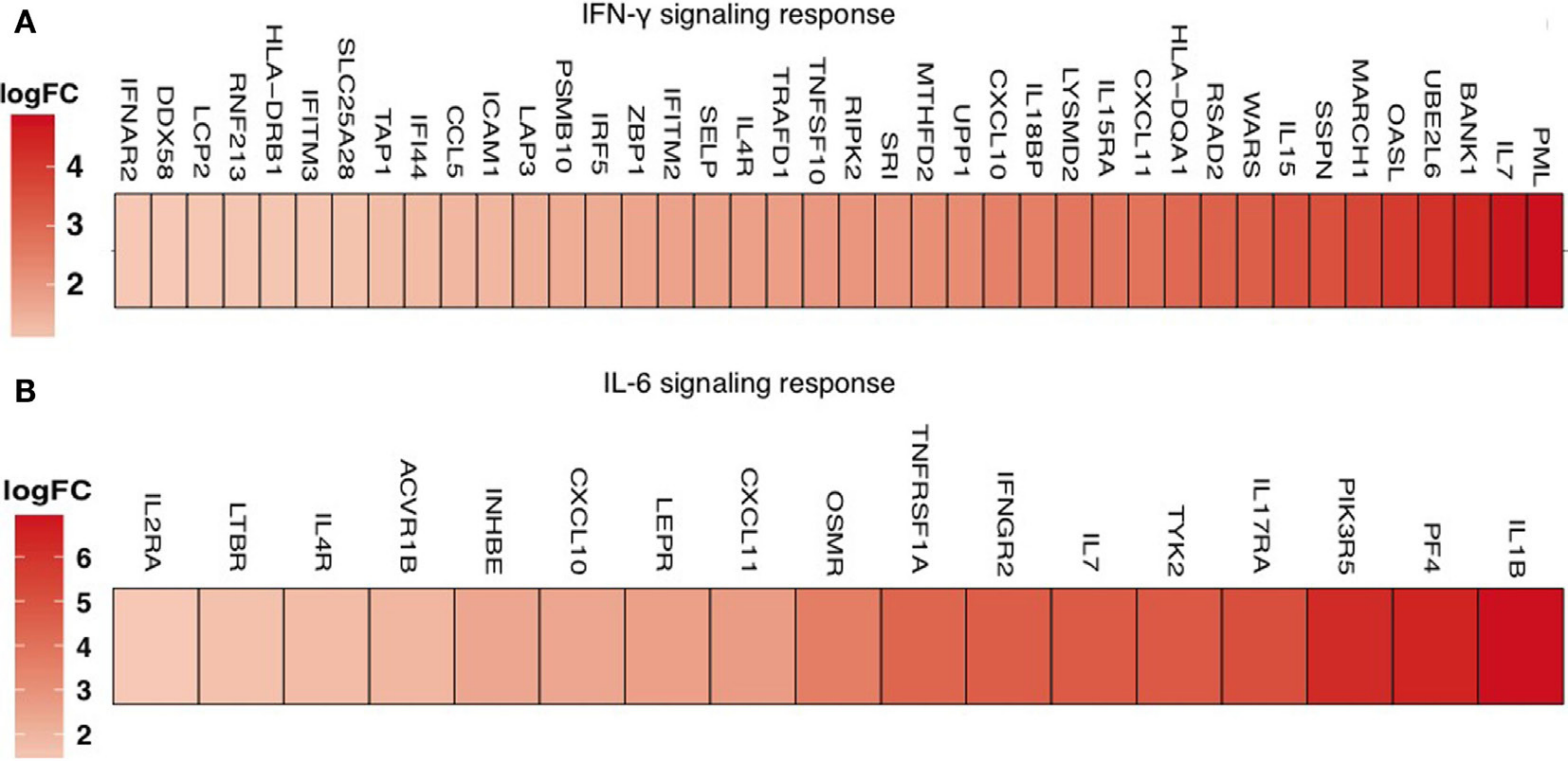
**Transcriptome profiling of CD4^+^ T cells in peripheral blood mononuclear cells shows upregulation of interferon-γ and IL-6 JAK-STAT responses in CD4^+^PD-1^+^ T cells of INR patients**. Enrichment of the IFN-γ response pathway (at FDR = 0.14) (above) and IL-6 JAK-STAT3 signaling pathway (below) in CD4^+^PD-1^+^ cells, identified by performing GSEA on the genes differentially expressed comparing INR (higher immune activation) to IR (less immune activation) among HIV/cART-treated patients. **(A)** Upregulation of the IFN-γ response pathway (at *p*-value <5% and FDR <15%) and **(B)** IL-6 JAK-STAT3 signaling pathway in CD4^+^PD-1^+^LAG3^−^ T cells, identified by performing GSEA on the genes differentially expressed comparing HIV-infected INR with IR patients. The color scale indicates the log fold-change of the gene being positively expressed and upregulated in the immune failure patients compared to the IR patients.

High level of immune activation is strongly associated with depletion of CD4^+^ T cells and increased proliferation of CD4^+^ T cells (41, 42). Expression of the nuclear antigen Ki67 is a recognized marker of cells that have recently been cycling and/or dividing. While higher expression of Ki67 negatively correlates with absolute CD4^+^ counts, it positively correlates with a larger size of the viral reservoir. While it is unclear, how increased proliferation and HIV reservoir size are linked, these data suggest that increased T-cell proliferation might also provide a mechanism for the maintenance of the HIV reservoir. Increased cycling and higher Ki67 protein expression further correlate with higher expression of PD-1, a marker that can be up-regulated as a result of homeostatic or antigen-induced proliferation, and T-cell exhaustion (43–46). There is a strong association between PD-1 and the immune activation marker Ki67 in CD4^+^ T cells from cART-treated individuals (29, 42). Consistent with these data, reduced PD-1 expression correlates with lower immune activation in HIV-elite controllers, the HIV^+^ infected individuals with immune control of viral loads (46, 47). Transcriptomic analyses of PD-1^+^ cells isolated from blood also suggest a significant increase in IFN-γ response genes and IL-6 response genes in INR with higher immune activation, compared to IR with lower immune activation (Figure 1). How PD-1 expression in CD4^+^ T cells contributes to HIV reservoir size and immune activation remains to be investigated. Notably, IL-6 and IFN-γ are found at increased levels in individuals with higher of immune activation (36, 40, 48). Future studies are required to examine the regulatory aspects of these cytokines in the context of immune activation, PD-1 expression, Th17 and T_reg_ cells in HIV^+^ patients. A recent study shows that activated CD4 T cells co-expressing PD-1, along with CCR7, CXCR5, and CCR6, may represent a highly functional population that is more susceptible to HIV infection and selectively lost in chronic HIV infection (49). Because 80% of the peripheral T cells reside in mucosal compartments such as gut (27, 50), which also constitutes a rich reservoir, it is critical to understand immune activation effects and the reservoir size, in the context of PD-1 expression and CD4^+^ T-cell homeostasis in the mucosa. Moreover, how immune activation impacts PD-1 expression on Th17 cells and T_regs_, and whether these cells harbor HIV reservoirs in the mucosa remain to be investigated.

## Alterations in Mucosal CD4 T-Cell Homeostasis during cART Treatment in HIV-Infected Patients

Intestinal epithelial barrier dysfunction causes an imbalance between the immune system, and mucosal repair and regeneration during primary HIV infection (51–54). Emerging evidence suggests that the disruption of gut mucosal CD4^+^ T-cell homeostasis, beyond just the depletion of CD4^+^ T cells, contributes to persistent systemic CD4^+^ T-cell activation and HIV pathogenesis in untreated and chronically treated HIV^+^ patients (9, 39, 55–57). However, CD4^+^ T-cell subsets in the gut and other mucosae are significantly different from CD4^+^ T-cell populations in peripheral blood in healthy individuals (58, 59). CD4^+^ T lymphocytes residing in the gut mucosa are predominantly of memory phenotype, and are prone to be more activated due to altered cytokine milieu, interactions with gut microbiota, and constant antigenic exposure (8, 25, 26). Gut CD4^+^ T cells also typically express the major HIV co-receptor CCR5, and the α4β7 integrin that promotes gut homing of T cells and can also facilitate HIV transmission (60, 61). The percentage of infected CD4^+^ T cells is much higher in gut mucosa than in circulation as HIV RNA is detectable in 60% of gut CD4^+^ T cells. By contrast, HIV RNA is detectable only in 0.01–1% of peripheral CD4^+^ T cells during acute infection (23). The underlying mechanisms for these differences are unclear. Unlike peripheral CD4^+^ T cells, the majority of reconstituted gut CD4^+^ T cells is central, transitional, and effector memory T cells, which are likely in a hyper-inflammatory state secreting cytokines that further stimulate HIV replication in patients treated with cART (27). Given these differences between peripheral and mucosal CD4^+^ T cells, and the contribution of CD4 imbalance to mucosal dysbiosis and systemic inflammation, it is important to gain a better understanding on CD4 T-cell homeostasis in the gut and other mucosa.

Most studies demonstrate a partial CD4^+^ T-cell restoration in HIV-infected individuals treated with cART (27). One study examined immune reconstitution in the gut using serial biopsies of rectosigmoid mucosa derived from cART-treated individuals. It revealed that HIV-mediated CD4^+^ T-cell depletion is more significant in immune-effector sites, such as the lamina propria compared to immune-inductive sites, such as the lymphoid tissue. Furthermore, longitudinal examination of individuals with acute HIV infection revealed that while CD4^+^ T-cell reconstitution by cART is complete in immune-inductive sites, it is only partial in mucosal immune-effector sites, compared with healthy HIV-uninfected controls (24, 62). While only limited studies have been performed to date in the context of acute and primary infection, evidence suggest that cART initiation early in the course of infection correlates to better gut CD4 T-cell reconstitution. In addition to changes in CD4^+^ T-cell numbers within the gut mucosa, altered trafficking of peripheral CD4^+^ T cells to gut has been proposed as a mechanism contributing to reduced CD4^+^ T-cell reconstitution in HIV-infected individuals undergoing treatment (23, 60, 61, 63).

While many of the HIV mucosal studies focus on gut/rectal mucosa, there are only a few studies that have examined CD4^+^ T cells in oral mucosa during SIV/HIV infection (64, 65). Although CD4^+^ T cells are depleted during SIV infection (66), it is not known whether persistent CD4^+^ T-cell perturbations post cART treatment contribute to immunopathogenesis in the oral mucosa. Oral opportunistic infections (67) and altered oral microbiome/mycobiome profiles (68–71) are important features of oral inflammation that have been linked to a wide range of pathologies, including periodontitis and oral cancer in HIV^+^ cART-treated patients (72–75). The oral microbiome comprises over 600 prevalent bacterial taxa and fungi, with distinct subsets predominating in different habitats (68, 70, 76–78). Similar to commensal dysbiosis and microbial translocation in the gut (79), perturbations in the oral microbiome, or opportunistic pathogenic infections, and associated inflammatory products (67, 70, 71, 80–86) may also contribute to oral immune dysregulation and HIV disease progression. While many of the microbial markers in the serum and plasma point to intestinal leakiness, it is not known whether these markers reflect microbial dysbiosis or systemic leakage of microbial products from the oral mucosa. We and others have begun to examine oral mucosa in HIV^+^cART-treated patients and in SIV infection, and found alterations in oral epithelial cells and CD4^+^ T cells (66, 87, 88). However, the underlying immune mechanisms of HIV-associated immune activation in the context of mucosal CD4^+^ T-cell profiles, HIV reservoirs, and immune restoration during chronic HIV disease are still unclear.

## Th17 Cell Changes in Mucosal Immune Pathogenesis

**Figure 2:**
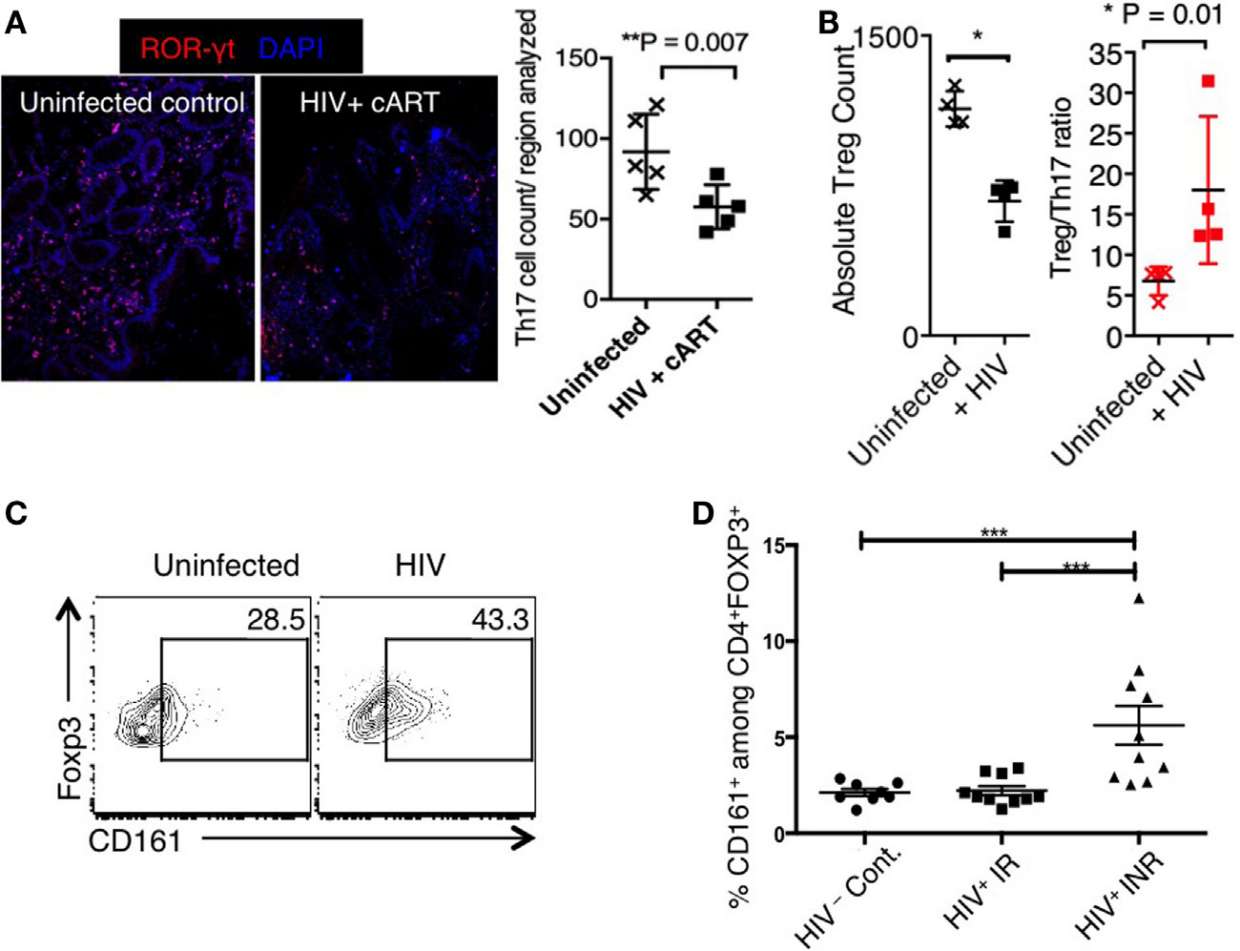
**(A)** Loss of Th17 cells in biopsies of transverse colon in HIV patients on cART. Frozen blocks of the biopsies were fixed, immunofluorescent stained using α-RORγt antibody (red) and 6-diamidino-2-phenylindole (DAPI) (nucleus; blue), and assessed by confocal microscopy. Confocal micrographs (left) and statistics (right). HIV infection induces T_reg_ cell loss **(B)**, but CD161 up-regulation in T_regs_ **(C)** in HTC. Three days after *in vitro* HIV infection, we stimulated the tonsillar cells using α-CD3 (T-cell receptor activation) and α-CD28 antibodies, and assessed the cells by flow cytometry 3 days later. Representative flow cytometric analyses show Foxp3^+^ T_reg_ cell count (left), and T_reg_/Th17 ratio (right) (gated on CD4^+^ cells) **(B)**, and CD161 expression in Foxp3^+^ cells **(C)**. **(D)** CD161 expression on FOXP3^+^ CD4 T cells in HIV-1 infected IR and INR patients. Shown are the frequencies of CD161^+^ cells gated on CD3^+^, CD4^+^, FOXP3^+^ CD127^−^CD25^+^ in 10 IR (Median age 47.8, 7M 3F, median CD4 count 910 c/ul), 10 INR (Median age 51.9, 7M 3F, median CD4 count 270 c/μl), and 8 HIV-uninfected healthy controls (HIV^−^Cont.). PBMCs were stained with the fluorochrome-conjugated antibodies, acquired by LSRII Fortessa and analyzed by flowjo. Anova test was used for multi-comparison analysis using graphPad Prism software. ***P < 0.0001.

Th17 cells are CCR6^+^, ROR-γt^+^, IL-17-producing CD4^+^ T cells that have a pivotal role in maintaining the epithelial barrier in the mucosa (89, 90). They play an important role in host defense against fungi and extracellular bacteria, and their importance is evident in mice and Autoimmune Polyendocrinopathy Candidiasis Ectodermal Dystrophy (APECED) patients (90–92). The protective roles of gut mucosal Th17 cells in HIV disease is becoming increasingly clear, and Th17 cell loss has been linked to loss of mucosal epithelial integrity, and results in multiple deleterious sequelae, including microbial translocation and gut inflammation (20, 93–95). Incomplete Th17 restoration in the gut despite long-term cART is also linked to persistence of immune activation (21, 23, 51, 93, 96–98). Fewer Th17 cells have been observed in the sigmoid colon of HIV-infected INR individuals (CD4 cell count <350 cells), compared to HIV-uninfected individuals. We also found in colon biopsies that the frequency of ROR−γt^+^ Th17 cells was substantially reduced in cART-treated HIV^+^ patients, compared to uninfected controls (Figure 2A). Previous studies have shown that CCR6^+^ memory and effector Th17 cells in both peripheral blood and inflamed tissues are preferential targets for HIV-1 infection (99). Though underlying mechanisms are unknown, more recent observations show specificities in HIV infection, where Th17 cells specific to Tetanus toxoid and *Candida albicans* were more permissive to HIV infection, than were CMV specific Th17 cells (99). These results may point to how specific cytokine milieu, or toll-like receptor (TLR) signaling components that differ with each infection, may determine the susceptibility of Th17 cells to HIV infection. While the loss of Th17 cells contributes to gut microbial translocation and systemic inflammation during HIV infection (20, 39, 63, 65, 93, 95, 100–105), the causes for incomplete Th17 cell restoration in the mucosa is unclear. In addition to the local effects on Th17 cells in lamina propria and MALT, perturbations in trafficking of Th17 cells can also alter Th17 homeostasis in the gut mucosa of HIV-infected persons (57, 63). For example, in INR patients, a significant increase in α4β7 positive peripheral Th17 lymphocytes positively correlates with integrated pro-viral DNA in rectum lymphoid cells compared to IR (106). Whether defective migratory capacities and increased HIV infection of gut Th17 cells contribute to impaired reconstitution of Th17 cells in the gut mucosa remain to be studied in different cohorts of HIV^+^ individuals. Specific components of the gut microbiome are known to stimulate the expression of cytokines in innate immune cells, which in turn can affect the generation and expansion of Th17 cells. Because gut microbiome is altered in HIV^+^ individuals (71, 79, 107), it is likely that it contributes to alterations in Th17 cell numbers and functions. Enhancement of microbiota using probiotics has been shown to modulate mucosal and systemic immune functions and improve GI tract immunity there by mitigating inflammatory sequelae, ultimately improving prognosis in HIV^+^ individuals (108). However, it remains to be seen whether the products of pathogenic microbes from co-infections, opportunistic commensals, differentially affect Th17 cell reconstitution in the gut. In our future studies, we will determine how inflammatory signals, such as microbial TLR ligands, affect Th17 cell viability in the context of their sensitivity to apoptosis and pyroptosis in mucosa and lymphoid tissues (REF).

## T_reg_ Changes in Mucosa

CD4^+^CD25^+^Foxp3^+^ T_regs_ are critical for immune balance and effective functioning of the immune system, both in normal and diseased states. They control inflammation by (1) producing immunosuppressive cytokines (109) and (2) inducing cytokine deprivation apoptosis of effector CD4^+^ T cells (110). They have therapeutic potential in many disease settings, such as infections, cancer, autoimmune diseases, and transplantation (109, 111–113). Severe autoimmunity and inflammation in the absence of T_regs_ in immune dysregulation, polyendocrinopathy, enteropathy, and X-linked inheritance (IPEX) patients and during mucosal infections (90–92), highlight the importance of T_regs_ in immune homeostasis. Because immune diseases are characterized by increase or decrease in numbers and function of T_regs_, there is considerable interest in identifying pathways that control the stability and viability of T_reg_ cells. The stability of Foxp3^+^ T_regs_ and generation of dysfunctional Foxp3^+^ cells at inflammatory disease sites also constitute an active area of immunology research. However, the functional effect of T_regs_ on HIV immune pathogenesis is poorly understood (114–116). Recent studies show that T_regs_ may not be detrimental to anti-HIV effector responses as previously thought. T_regs_ directly inhibit HIV-1 replication in activated T cells (117), and do not suppress antigen specific anti-HIV CD8 responses (47, 118). Moreover, T_regs_ in circulation strongly correlate with decreased generalized T-cell activation (47, 100–102, 119–129), showing that they may also play critical roles in mitigating immune hyper-activation. On the contrary, some studies suggest that T_regs_ have a role in suppressing immune response to HIV and mucosal pathogens (130). Absence of markers to distinguish natural and induced FOXP3^+^ cells, functional and dysfunctional T_regs_, and limitations in assessing functions of FOXP3^+^T_regs_ partly play a role in generating these discrepancies (65, 120, 130–132). Given that FOXP3 and CD25 are expressed transiently on human effector cells, another important caveat is the sole usage of FOXP3 or CD25 as T_reg_ markers in most HIV studies.

Although HIV infection causes depletion of CD4^+^ T_regs_ leading to their lower absolute cell numbers in blood and gut mucosa (133), FOXP3^+^ T_regs_ are observed in increased proportions in relation to Th17 cells in gut mucosa and oral mucosa during SIV/HIV infection (64, 65, 93, 134). Given the reciprocal relationship between Th17 cells and T_regs_, T_reg_/Th17 ratio may be more important than the absolute levels of either one of the subsets independently (21, 94, 103, 131, 132, 135–139). Consistently, increased T_reg_/Th17 cell ratio correlates to more advanced disease in immune non-responders (CD4 <350 cells/μl), viral load, plasma levels of sCD14, sCD163, and IL-1RA (markers of monocyte activation), as well as increased T-cell activation (93, 96, 104, 116, 138, 140–142). However in HIV^+^ patients with elevated levels of immune activation, it is not clear whether the T_regs_ are functional or dysfunctional, or natural or induced (130, 132, 136, 143). Our data also show that HIV-infected oral tonsillar cells show an increase in T_reg_/Th17 ratio (Figure 2B). Increased frequencies of T_regs_ can be attributed to indolemine 2,3 dioxygenase (IDO) produced by plasmocytoid DCs, which can promote T_reg_ induction during HIV-1 infection (144). We hypothesize that increased proportions of T_regs_ may also be attributed to preferential apoptosis or pyroptosis of conventional CD4^+^ T cells, including Th17 cells in the mucosa. We have previously shown that conventional CD4 T effector cells are highly susceptible to FAS-mediated apoptosis compared to T_regs_ *in vitro* and *in vivo* (145). In our future studies, we will examine whether differential sensitivities of Th17 cells and T_reg_ cells to FAS mediated apoptosis and, or pyroptosis, also contribute to increased T_reg_/Th17 ratio in mucosa during HIV infection. We will also determine whether T_regs_ are just altered in proportions, and if increase in the dysfunctional Foxp3^+^ cells contributes to HIV disease progression in the immune activation scenario.

## Are T_regs_ Dysfunctional in HIV Disease?

Emerging evidence shows that T_regs_ become dysfunctional and acquire capacity to produce inflammatory cytokines (146–149), despite the expression of FOXP3, during infections and inflammatory diseases. Such pro-inflammatory cytokine producing poly-functional FOXP3^+^ cells are shown to have lost their suppressive capacities in the context of certain diseases, such as psoriasis and inflammatory bowel diseases (146, 147, 149). Our previous findings show that TLR-2 signaling in the context of IL-6 induces pro-inflammatory IL-17A production in T_regs_ transiently during an oral mucosal infection. While transient increase of IL-17A producing T_regs_ during an acute infection is not detrimental, the persistence of pro-inflammatory cytokine producing T_regs_ is strongly associated with inflammation (146, 149, 150). Similarly during HIV infection, pro-inflammatory cytokines, such as IL-6, commensal bacteria, and their metabolites may affect FOXP3^+^ cells, induce T_reg_ plasticity, and trigger an increase in dysfunctional T_regs_ (146, 147, 149). For example, IL-6 and soluble IL-6 receptor are observed at increased levels in cART-treated HIV patients, especially in those with a history of immune restoration disease (36). Increased IL-6 levels may contribute to T_reg_ dysregulation that may further lead to the vicious loop of exacerbating mucosal and systemic immune dysbiosis in these patients. Another parameter that could affect T_reg_ stability is TLR signaling. Excessive TLR signaling in the context of immune activation may also control T_reg_ expansion and plasticity during HIV infection. HIV-1 infection is known to modulate TLR responses by altering TLR expression and activation levels, and regulating responses of innate immune cells to TLR stimulation, which may subsequently affect immune activation levels. Given the prevalence of microbial co-infections in HIV^+^ patients [e.g., *Mycobacterium tuberculosis* (151–153), *Porphyromonas gingivalis* periodontitis (67, 154), and *Candida* infections (75, 83)], increased soluble TLR expression and signaling may also arise from these opportunistic infections. These co-infections can cause an increase in dysregulated T_regs_, and sustain chronic inflammation during HIV-1/SIV infection. Consistent to this hypothesis, the percentage of TLR-2- and TLR-4-expressing T_regs_, and the levels of TLR-9 signaling are significantly increased in HIV^+^ patients with CD4 cell counts <500 cells/ml in whole blood, correlating with their immune activation (132, 155, 156). Further studies are required to determine how co-infections and TLR signals contribute to homeostasis and dysfunction of FOXP3^+^ T_regs_ in the mucosa.

Pro-inflammatory cytokine producing FOXP3^+^ cells have been associated with immune dysregulation, and can be identified by a recently described novel marker, CD161 (147, 149). CD161 also defines a Th1/Th17 poly-functional subset of resident memory T lymphocytes (157, 158). In the context of autoimmunity, pathogenic CD161^+^ memory cells are resistant to T_reg_-mediated suppression, which may be another mechanism of loss of immune homeostasis (159). We observed that HIV infection of tonsillar cells induced CD161 expression in T_regs_ (23 ± 6 versus 44 ± 5%, *P* < 0.05) (Figure 2C). These results support the possibility that HIV infection may contribute to increased CD161 expression and T_reg_ dysfunction in mucosa. Consistent to these data, we also found that a higher proportion of FOXP3+ cells were CD161 positive in INR (with higher immune activation), compared to IR patients (with lower immune activation) in the peripheral blood (Figure 2D). How IL-6 and TLR-ligands enhance CD161 expression, and how CD161 expression contributes to T_reg_ dysfunction during HIV infection are areas of intense investigation in our laboratories. In addition to FOXP3+ T_regs_, FOXP3 negative T-regulatory type-1 (Tr1) cells are major producers of IL-10, and may have a beneficial role in controlling immune activation in early HIV infection (160). However, a recent cross-sectional study in patients with non-progressive HIV-1 infection showed that concentrations of TGF-β1 and IL-10 are significantly decreased in their plasma, while IL-1β, IL-12p70, and TNF-α are increased, compared to patients with progressive infection (161). Another study also showed that the protein levels of IL-1β, IL-6, and IL-10 were significantly lower in plasma of HIV-1-exposed seronegative individuals than HIV-1-infected patients. These data show that disease and infection progression are associated with increased IL-10 and basal pro-inflammatory responses (162). The relationship between IL-10 and Foxp3^+^ T_regs_ remains to be seen. Taken together, since induction, stability, and functions of FOXP3^+^ T_regs_ largely depend on the cytokine milieu, detailed studies focusing on T_reg_ plasticity and dysfunction in HIV^+^ patients with distinct cytokine will significantly improve our understanding of immune dysregulation in HIV these patients (109, 163, 164). Such studies will ascertain if the reduction of Treg dysfunction will mitigate HIV-associated immune activation, which in turn would also result in fewer susceptible target CD4^+^ T cells, and an environment that prevents efficient HIV replication *in vivo*.

## Therapeutic Interventions Modulating Th17 Cells and T_regs_

Despite the development and optimization of cART that successfully suppresses HIV replication in majority of HIV^+^ patients, a treatment that can cure HIV disease is not yet available. It is unlikely that one single approach will lead to a cure for AIDS. The interaction between HIV and CD4^+^ T cells is complex and involves contrasting effects with respect to virus replication (165). On the one hand, CD4^+^ T cells serve as mediators of antiviral immune responses. It has also been reported that depletion of CD4^+^ T cells prior to SIV infection in rhesus macaques in fact is associated with higher viral loads, expansion of pro-inflammatory monocytes, and massive activation and infection of macrophages and microglia that appear to be the predominant population of productively infected cells (141). These data highlight the protective roles of CD4^+^ T cells in modulating inflammation and reducing the viral burden. On the other, proliferating CD4^+^ T cells are main targets for infection and viral replication. Residual inflammation promotes HIV reservoir persistence by triggering the infection of susceptible cells, and both these processes are inextricably interrelated in a vicious cycle. Therefore, therapies should be targeted to reduce immune activation and inflammation and HIV persistence, as well as enhancing antiviral functions. A previous study has shown that interleukin-21 (IL-21) treatment restores not only the Th17 cells in the gut mucosa, but also dramatically reduces immune dysfunction in rhesus macaques (104, 141, 142). IL-2 had been employed as a cART adjuvant in phase III clinical trials, but did not restore gut mucosal CD4^+^ T cells (166), failing to confer any clinical benefit. Despite successful CD4^+^ T-cell expansion in peripheral blood, IL-2 also expanded T_reg_ like CD25^+^ cells, increased the levels of IL-6 and D-dimer, inflammation, and activation of the coagulation cascade (167, 168). Whether the expanded T_reg_-like cells were FOXP3^+^, or were dysfunctional, was not assessed in those studies. Recombinant human IL-7 (r-hIL-7) has emerged as another candidate immune-based therapy that could succeed in expanding T cells and inducing the expression of gut homing receptor α4β7, without expanding T_reg_ cells and induction of pro-inflammatory cytokines during administration (57, 169). Phase I studies have demonstrated the effect of r-hIL-7 on expansion of T cells (57, 170) and suppressing colonic and systemic inflammation in chronic HIV infection. While IL-7 is a gamma-chain cytokine that could promote T_reg_ survival (171), the direct impact of r-hIL-7 treatment on Th17 cells and T_regs_ have not been addressed to date in HIV^+^ patients.

## Conclusion

**Figure 3:**
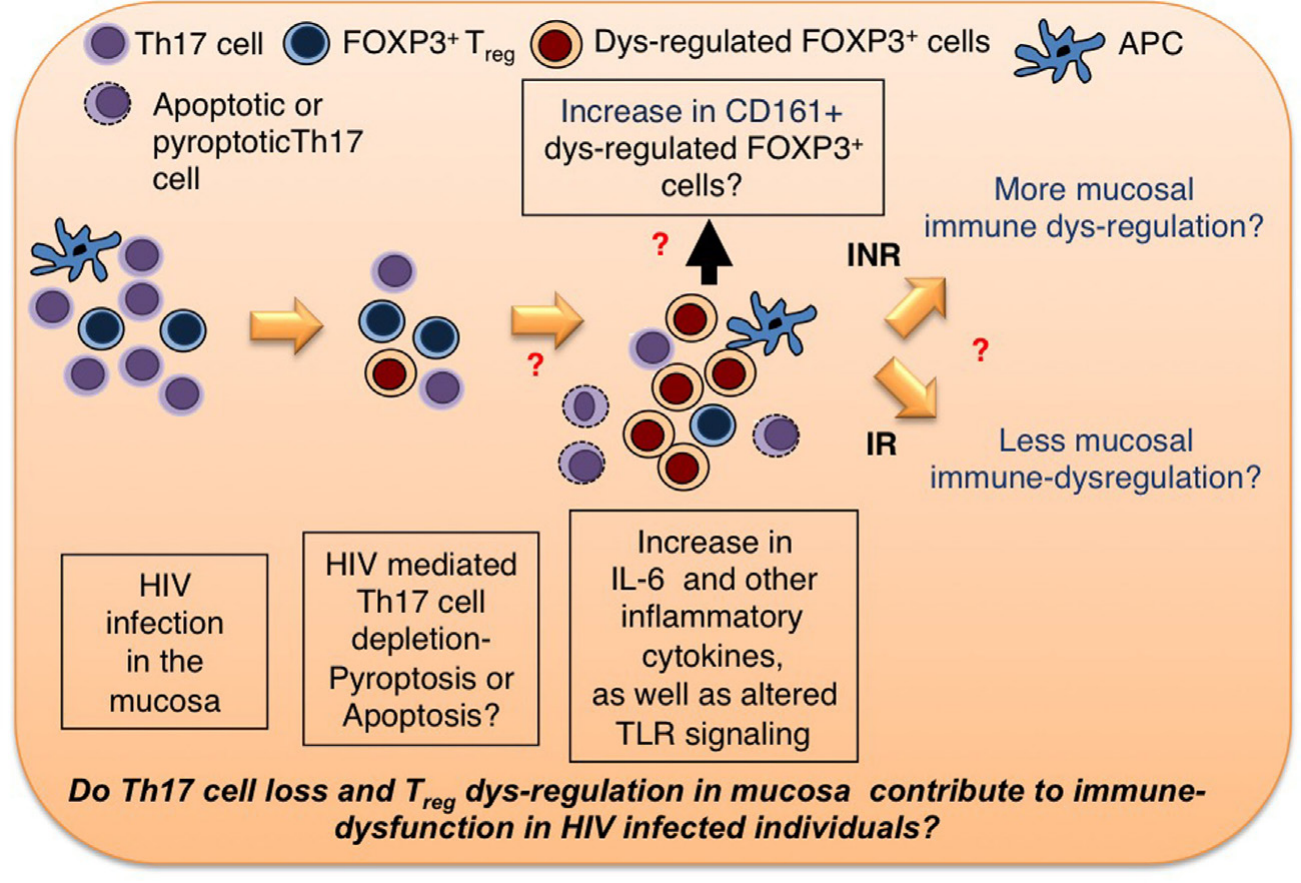
**Th17 cell and T_reg_ homeostasis in mucosa**. HIV infection leads to cellular loss of Th17 cells and T_regs_ in mucosa. The questions that need to be addressed are as follows: (1) Do inflammatory cytokines and perturbations in TLR signaling contribute to increase in dysregulated FOXP3^+^ cells (T_regs_?)? If so, how? What are the markers of dysregulated T_regs_? (2) Does T_reg_ dysregulation contribute to persistent immune dysfunction in INR HIV^+^ patients? We hypothesize that therapeutic strategies reducing dysfunctional T_regs_ and increasing protective Th17 cells in the mucosa should be employed as a part of synergistic approach to cure HIV disease.

Residual inflammation can be treated by finding synergies between different approaches that are aimed to restore mucosal Th17 cells, and reversing T_reg_ dysfunctions in HIV^+^ patients. Given the (1) significant differences between mucosal T cells and circulating T cells, (2) immediate interactions of mucosal T cells with the microbiome, (3) gut microbial dysbiosis in HIV^+^ patients, (4) the ability of Th17 cells to maintain mucosal barrier integrity, and a pronounced loss of Th17 cells in HIV^+^ patients, and (5) ability of T_regs_ to control immune activation, and the possibilities for them to become dysfunctional in HIV^+^ patients, more research should be devoted to mucosal Th17 cells and T_regs_, and their contribution to systemic immune activation in HIV-infected individuals. A better comprehension of these cells will shed light on HIV-mediated mucosal immune dysfunction, and possible new interventional strategies to restore their functions. Because we hypothesize and present *in vitro* data showing that HIV infection in the context of inflammatory milieu may contribute to dysregulation of these two lymphocyte subsets in the mucosa (Figures 2 and 3), we believe that anti-inflammatory therapeutic strategies increasing protective Th17 cells in the mucosa should be employed as a part of synergistic approach to cure HIV disease.

## Methods

### Human Tonsillar Cultures

We obtained tonsils from the discarded tissues from HIV-negative patients who undergo tonsillectomy surgery at University Hospitals/Case Western Reserve University (Age = 8–16) and infected the bulk human tonsillar cultures (HTC) with replication-competent HIV-1 NLAD8-GFP virus stocks (30 ng p24/10^6^ cells) that were generated by transfecting HEK293T cells with pro-viral DNA. NLAD8-GFP was derived from NL43-GFP-IRES-Nef (172) by replacing the CXCR4-tropic envelope with CCR5-tropic AD8 envelope. The resulting construct expresses GFP and Nef on a bi-cistronic mRNA (173, 174).

### Study Cohort and Design

This study included 17 HIV-infected subjects on c-ART for 2 years or greater. The individuals were categorized into two groups – 10 IR who had high CD4^+^ T-cell counts (CD4 >500 cells/mm^3^) and 7 INR who had low CD4^+^ T cells despite viral suppression (CD4 >350 cells/mm^3^). CD4^+^ T cells from these subjects were sorted for cell surface expression of CD3^+^, CD4^+^, CD45RO^+^, CD45RA^−^, and PD-1 in the absence of LAG3, characterized as PD-1^+^LAG3^−^ cells. One thousand cells were sorted for Illumina RNA-Sequencing.

### RNA-Seq Pre-Processing

Pre-processing of the sequencing data was performed by integrating open source tools and R-Bioconductor packages. The raw reads were trimmed off any adaptor sequence contaminants using Trimmomatic 0.32, followed by mapping trimmed reads onto the Ensembl version of the Human Genome (Grch38) using the STAR 2.4.0f1 aligner. The transcript counts were then estimated by using HTSeq. The transcript expression was then normalized by trimmed mean of M-values (TMM). Any outlier samples based on abnormalities in gene expression were removed.

### Transcriptomic Profiling of RNA-Seq Data

The differences in gene expression profiles comparing INR to IR in the CD4^+^PD-1^+^LAG3^−^ T cells was determined by fitting a generalized linear model (GLM) for every transcript expression. The transcript expression was used as the dependent variable and the groups of interest as the independent variable. Differentially expressed genes were identified by using a likelihood ratio test to test if the fold changes are different from 0. Pathways enriched among the differential expressed genes were identified by Gene Set Enrichment Analysis (GSEA) pre-ranked by the decreasing order of −log10(*p*-value) × sign(log-fold change) of the gene with a 1000 permutations. The pathway database used was the Hallmark genes (version 5.0) from the Molecular Signatures DataBase (MSigDB). The obtained *p*-values were corrected for multiple comparisons by the Benjamini and Hochberg method.

## Consent Procedure

The study staff will talk with the volunteers about the consent information. Study participants are free to ask questions about this study at any time. If they agree to take part in this study, they will be asked to sign the consent form. They will get a copy to keep. Before they learn about the study, it is important that they know the following:

- Their participation is entirely voluntary.
- They may decide not to take part in or to withdraw from the study at any time without losing the benefits of their routine medical care.

Discarded tonsils are collected from minors involved in tonsillectomy surgery. Otherwise minors are not directly involved in the study.

## Ethics Statement

Human cells were obtained from PBMC under approved protocols, reviewed and approved by the University Hospitals Case Medical Center Institutional review boards.

## Author Contributions

PP designed the study, performed experiments, analyzed data and wrote the manuscript; SAY, NB, DM, AT and SPR performed experiments; ADL helped with providing gut biopsies and contributed to discussions, RPS contributed to discussions and edited the manuscript, and AW edited the manuscript.

## Conflict of Interest Statement

The authors declare that the research was conducted in the absence of any commercial or financial relationships that could be construed as a potential conflict of interest.
